# Acute kidney injury in major abdominal surgery: incidence, risk factors, pathogenesis and outcomes

**DOI:** 10.1186/s13613-018-0369-7

**Published:** 2018-02-09

**Authors:** Joana Gameiro, José Agapito Fonseca, Marta Neves, Sofia Jorge, José António Lopes

**Affiliations:** Division of Nephrology and Renal Transplantation, Department of Medicine, Centro Hospitalar Lisboa Norte, EPE, Av. Prof. Egas Moniz, 1649-035 Lisbon, Portugal

**Keywords:** Acute kidney injury, Postoperative, Incidence, Prognosis, Risk factors, Pathogenesis

## Abstract

Acute kidney injury (AKI) is a common complication in patients undergoing major abdominal surgery. Various recent studies using modern standardized classifications for AKI reported a variable incidence of AKI after major abdominal surgery ranging from 3 to 35%. Several patient-related, procedure-related factors and postoperative complications were identified as risk factors for AKI in this setting. AKI following major abdominal surgery has been shown to be associated with poor short- and long-term outcomes. Herein, we provide a contemporary and critical review of AKI after major abdominal surgery focusing on its incidence, risk factors, pathogeny and outcomes.

## Background

Acute kidney injury (AKI) is a common occurrence in hospitalized patients and it has a detrimental effect on patient outcome. Indeed, AKI is associated with increased costs, length of hospital stay and in-hospital mortality [1–3]. Postoperative AKI has been associated with higher risk of developing chronic kidney disease (CKD) [4, 5] and increased early [6–17] and long-term mortality [10–22], comparable to the consequences of AKI facing critically ill patients. Postoperative AKI is hence of particular interest, serving as a measurable indicator of perioperative harm and an important potential target for intervention [23].

The clinical characteristics and the impact of AKI in cardiac surgery have been extensively studied [24, 25], and most of the published data regarding AKI in the noncardiac surgery population are limited to high-risk aortic procedures [26–31]. Abdominal surgery is frequently associated with AKI. Recently, a number of studies have addressed AKI following major abdominal surgery [11, 19, 32, 33], especially since it shows a pathophysiology that is distinct from that of cardiac and vascular surgery. Therefore, it is unsuitable to assume that the risk factors for AKI after abdominal surgery are the same as those after cardiac and vascular surgery. The purpose of this review is therefore to perform a critical and contemporary review of the incidence, risk factors, pathogenesis and outcome of AKI in patients undergoing major nonvascular abdominal surgery.

## Incidence, risk factors and pathogenesis

### Incidence

Over the last decade, the definition of AKI has evolved from the former term acute renal failure to a set of uniform criteria combining small changes in creatinine and urine output ultimately defining AKI [34]. The first definition of AKI, the Risk, Injury, Failure, Loss of kidney function and End-stage kidney disease (RIFLE) classification, was published in 2004 [35]. In 2007, the Acute Kidney Injury Network (AKIN) classification, also known as ‘modified RIFLE’, was published [36]. In recent times, the RIFLE and AKIN classifications have been merged into the Kidney Disease: Improving Global Outcomes (KDIGO) classification in order to provide simpler and more integrated criteria applicable in clinical activity, research, and public health surveillance. (Table 1) [37] AKI is thus defined as an increase in serum creatinine (SCr) by ≥ 0.3 mg/dl (≥ 26.5 μmol/l) within 48 h; or an increase in SCr to ≥ 1.5 times the baseline value, which is known or presumed to have occurred within the prior 7 days; or urine volume < 0.5 ml/kg/h for 6 h [38]. These classifications also categorize patients according to the severity of AKI [38].

**Table 1:** Risk, Injury, Failure, Loss of kidney function, End-stage kidney disease (RIFLE) [35], Acute Kidney Injury Network (AKIN) [36], and kidney disease improving global outcomes (KDIGO) [37] classifications

Class/stage	SCr/GFR			UO		
	RIFLE	AKIN	KDIGO	RIFLE	AKIN	KDIGO
Risk/1^a^	↑ SCr X 1.5 or ↓ GFR > 25%	↑ SCr ≥ 26.5 μmol/l (≥ 0.3 mg/dl) or ↑ SCr ≥ 150–200% (1.5–2X)	↑ SCr ≥ 26.5 μmol/l (≥ 0.3 mg/dl) or ↑ SCr ≥ 150–200% (1.5–2X)	<0.5 ml/kg/h (> 6 h)	<0.5 ml/kg/h (> 6 h)	<0.5 ml/kg/h (> 6 h)
Injury/2^a^	↑ SCr X 2 or ↓ GFR > 50%	↑ SCr > 200–300% (> 2–3X)	↑ SCr > 200–300% (> 2–3X)	<0.5 ml/kg/h (> 12 h)	<0.5 ml/kg/h (> 12 h)	<0.5 ml/kg/h (> 12 h)
Failure/3^a^	↑ SCr X 3 or ↓ GFR > 75% or if baseline SCr ≥ 353.6 μmol/l (≥ 4 mg/dl) ↑ SCr > 44.2 μmol/l (> 0.5 mg/dl)	↑ SCr > 300% (> 3X) or if baseline SCr ≥ 353.6 μmol/l (≥ 4 mg/dl) ↑SCr ≥ 44.2 μmol/l (≥ 0.5 mg/dl) or initiation of renal replacement therapy	↑ SCr > 300% (> 3X) or ↑SCr to ≥ 353.6 μmol/l (≥ 4 mg/dl) or initiation of renal replacement therapy	<0.3 ml/kg/h (> 24 h) or anuria (> 12 h)	<0.3 ml/kg/h (24 h) or anuria (12 h)	<0.3 ml/kg/h (24 h) or anuria (12 h)

*SCr* serum creatinine, *GFR* glomerular filtration rate, *UO* urine output, *RIFLE* Risk, Injury, Failure, Loss of kidney function (dialysis dependence for at least 4 weeks), End-stage kidney disease (dialysis dependence for at least 3 months), *AKIN* Acute Kidney Injury Network, *KDIGO* kidney disease improving global outcomes

^a^Risk class (RIFLE) corresponds to stage 1 (AKIN and KDIGO), injury class (RIFLE) corresponds to stage 2 (AKIN and KDIGO), and failure class (RIFLE) corresponds to stage 3 (AKIN and KDIGO)

In the past decades, the incidence of AKI has suffered an increase and has been related to multiple factors such as an increasingly aging population, increasing number of comorbidities of the hospitalized population, increased prevalence of chronic kidney disease and diabetes, and the liberal use of intravenous contrast agents for imaging and cardiovascular intervention procedures [39].

Additionally, mortality has been trending downwards despite the reported modifications in the clinical profile and characteristics of patients with AKI [40, 41]. Nonetheless, it is not clear if this fact can be credited to an improvement in patient care or to specific interventions or therapies directed at those with AKI [42, 43].

Depending on the classification system employed in the studies, the reported incidence of AKI varies from 5.0 to 7.5% in hospitalized patients, reaching up to 50–60% in critically ill patients [2, 44–46].

Surgery remains a leading cause of AKI in hospitalized patients, accounting for up to 40% of in-hospital AKI cases. The incidence of AKI in this group of patients is variable, depending on the surgical setting and the AKI definition used, with the highest rates found after cardiac (18.7%), general (13.2%), and thoracic (12.0%) surgeries [47, 48].

A considerable heterogeneity regarding the rate of AKI reported has been shown in recent studies of AKI following major abdominal surgery. (Table 2) The incidence varied between 3.1 and 35.3%, with the majority of patients in all studies placing in the less severe stage of AKI (Risk or Stage 1). One of the major limitations of these studies is that, only three evaluated simultaneously serum creatinine and urine output to define and categorize AKI, as recommended [35].

**Table 2:** Incidence and categorization of AKI and its association with mortality after major abdominal surgery

Study	Design	Setting	Criteria	AKI definition	N	Incidence	Mortality	AUROC
Armstrong et al. [59]	Retrospective, single center	HBP	SCr	AKIN	1535	5.10% 1–4.0% 2–0.8% 3–0.3%	1.7% AKI versus 3.4% non-AKI, *P* = 0.21	NA
Bell et al. [58]	Interrupted time series analysis	MA/GI	SCr	KDIGO	3271	9.80%	NA	NA
Bihorac et al. [20]	Retrospective, single center	MA/GI	SCr	RIFLE	2337	39.3%	NA	NA
Biteker et al. [12]	Prospective, single center	MA/GI	SCr	RIFLE	510	6.7%	6.1% AKI versus 0.9% non-AKI, *P* = 0.003	NA
Brunelli et al. (2012)	Retrospective, single center	MA/GI	SCr	AKIN/RIFLE	1912	26.80%	NA	NA
Causey et al. [32]	Retrospective, single center	Colorectal	SCr	RIFLE	339	11.8%	6.30% AKI versus 0.9%, *P* = 0.065	NA
Chao et al. (2013)	Prospective, multicenter	MA/GI	SCr	AKIN	4240	23.1% 1–13.7% 2–1.8% 3–7.6%	28.40% 1–16% 2–29.7% 3–48.3% (HR 3.19, 95% CI 2.16–4.71; *P* < 0.001)	0.728
Cho et al. [4]	Prospective, single center	HBP	SCr, UO	AKIN	131	7.6% 1–3.8% 2–1.5% 3–2.3%	7.10% AKI versus 2.5% non-AKI, *P* > 0.05	NA
Coca et al. [98]	Retrospective, multicenter	Non cardiac surgery	SCr	AKIN	11.460	18.9% 1–5.2% 2–2.5% 3–1.2%	NA	NA
Correa-Gallego et al. [60]	Retrospective, single center	HBP	SCr	RIFLE	2166	15.5% R 12.8% I 2.3% F 0.4%	1% AKI versus 2% non-AKI, *P* = 0.5	NA
Grams et al. [89]	Retrospective, single center	MA/GI	SCr	KDIGO	44.597	13.2% 1–9.4% 2–2.2% 3–1.5%	IRR 6.40 (95% CI, 5.75, 7.12) *P* < 0.05)	NA
Kambakamba et al. [67]	Retrospective, single center	HBP	SCr	AKIN	829	8.2%	21% AKI versus 0.3% non-AKI, P < 0.001	0.765
Kim et al. [68]	Retrospective, single center	UGI	SCr	KDIGO	4718	14.4% 1–12.5% 2–1.3% 3–0.6%	3.8% AKI versus 0.3% non-AKI, *P* < 0.001 (OR, 8.75; 95% CI, 3.98–19.27; *P* < 0.001)	NA
Lee et al. [62]	Retrospective, single center	UGI	SCr	AKIN	595	35.3% 1–30.3% 2–2.7% 3–4.2%	4.80% AKI versus 2.1% non-AKI, *P* = 0.115	NA
Slankamenac et al. [64]	Retrospective, single center	HBP	SCr, UO	RIFLE	569	15.1%	22.5% AKI versus 0.8% non-AKI, *P* < 0.001	0.75
Sun et al. [69]	Retrospective, single center	GYN	SCr	AKIN	863	3.1%	NA	NA
Sun et al. [69]	Retrospective, single center	MA/GI	SCr	AKIN	1351	9.6%	NA	NA
Teixeira et al. [8]	Retrospective, single center	MA/GI	SCr, UO	KDIGO	450	22.4% 1–63.4% 2–19.8% 3–16.8%	20.8% AKI versus 2.3% non-AKI, *P* < 0.001; OR 3.7, 95% CI 1.2–11.7, *P* = 0.024	NA
Tomozawa et al. [65]	Retrospective, single center	HBP	SCr	AKIN	642	12.1% 1–9.8% 2–2.0% 3–0.3%	14.1% AKI versus 2.3% non-AKI, *P* < 0.0001	NA
Vaught et al. [9]	Retrospective, single center	GYN	SCr	RIFLE	2341	12.6% R–7.9% I–2.7% F–1.9%	10% AKI versus 0.5% non-AKI, *P* < 0.0083	0.88

*GI* gastrointestinal, *HPB* hepato-biliary, *RIFLE* risk, injury failure, loss, end stage, *AKIN* Acute Kidney Injury Network, *KDIGO* Kidney Disease Improving Global Outcomes, *MA* major abdominal, *GYN* gynecological, *SCr* serum creatinine, *UO* urinary output, *IRR* incidence rate ratio, *NA* not available

Urine output (UO) is a sensitive and early marker for AKI, independent of serum creatinine, thereby included as a criterion to diagnose AKI [49, 50]. However, recent literature reports that there is a physiologic reduction in UO as a result from hypovolemia, anesthesia and release of aldosterone and vasopressin in response to stress, which raises the hypothesis that UO may not be a reliable criterion for postoperative AKI, or that the threshold for AKI diagnosis with UO should be lower [51–53].

Research has focused on serum and urine biomarkers that could predict AKI before functional damage occurs [54]. This has been investigated mainly in cardiac procedures, with the most promising marker being plasma and urinary neutrophil gelatinase-associated lipocalin (NGAL) [54]. Also, the combination of urinary Kidney Injury Molecule-1 (KIM-1), *N*-acetyl-beta-d-glucosaminidase, and NGAL improved the sensitivity of early recognition of postoperative AKI when compared with individual biomarkers [55]. Recently, tissue inhibitor of metalloproteinases-2 (TIMP-2) and insulin-like growth factor binding protein 7 (IGFBP7) have been validated as risk predictors for AKI [56].

According to a recently published meta-analysis of 19 studies representing 82,514 patients undergoing abdominal surgery, the pooled incidence of AKI was 13.4% [23]. However, the incidence did not significantly vary by AKI definition, surgical category or inclusion or exclusion of preexisting CKD, demonstrating that other factors are probably also implied, such as the different surgical settings and baseline patient characteristics between individual studies [23].

### Risk factors

A number of studies have investigated and identified patient- and procedure-related risk factors associated with the development of AKI, namely older age, African American race, hypertension, diabetes mellitus and CKD [20, 48]. Patient-related factors are often more strongly associated with postoperative mortality than surgical factors [57].

Focusing on major abdominal surgery, demographic patient characteristics such as male gender, older age, and higher body mass index, as well as preexisting CKD, hypertension, cardiovascular disease, diabetes, chronic obstructive pulmonary disease, metastatic cancer, hypoalbuminemia, use of angiotensin-converting enzyme inhibitors (ACEI) or angiotensin-receptor blockers have been implicated as predisposing to AKI [8, 9, 58–65].

Additionally, several risk assessment scores have been associated with higher incidence of AKI. A higher MELD score, which predicts liver failure progression; a higher Revised Cardiac Index score, developed to predict cardiac complications and mortality after major noncardiac surgery; and higher SAPS II score, used to evaluate disease severity, have all been independently associated with AKI [8, 63, 65, 66].

Numerous studies have established the negative bearing of surgery or procedure-related factors in AKI in major abdominal surgery, specifically the use of intravenous contrast for vascular imaging and intervention, the use of diuretics and vasopressors, more invasive procedures, episodes of intraoperative hemodynamic instability, need for intraoperative blood transfusions, large colloid infusion during surgery, epidural anesthesia in liver resections and cases of emergent surgery [8, 9, 58, 60–63, 65, 67–69].

Nevertheless, the impact of the urgency of surgery has not been consensual in all studies. For instance, urgent surgery was not associated with an increased risk of postoperative AKI in a recent study by Teixeira et al. [8], despite the higher incidence of risk factors for AKI in these patients.

The role of laparoscopy has also been studied as the creation of a pneumoperitoneum is concomitant to increased intraabdominal pressure and the associated hormonal modifications that have been associated with decreased renal blood flow and could be linked to AKI [8]. Nevertheless, Teixeira et al. [8] demonstrated no difference in AKI between patients undergoing laparoscopy versus laparotomy.

O’Connor et al. [23] essayed to determine AKI incidence in different surgical settings, namely gastrointestinal, upper gastrointestinal, hepato-biliary, colorectal and major gynecological surgeries, however they were not able to demonstrate a significant difference in pooled AKI between these subgroups due to substantial heterogeneity between the studies. Similarly, in the study by Teixeira et al., colorectal surgery had an increased rate of AKI, which was not evidenced in other surgery types such as gastric, hepato-biliary and pancreatic, small bowel and esophageal. However, this finding was not independently associated with a higher risk of postoperative AKI [8]. These studies did not analyze the incidence of AKI after liver transplant surgery which can reach up to 70%, as it includes several specific risk factors in its pathogenesis, namely those related to the recipient and graft [70, 71]. Also important to consider, with the increasing prevalence of obesity in the global population, the prevalence of bariatric surgery has risen in the past decades and AKI has also been reported in 5–10% of these patients [72, 73].

Growing evidence has demonstrated that the need for intraoperative blood transfusions may contribute to organ injury in susceptible patients by promoting a pro-inflammatory state, exacerbating tissue oxidative stress, and activating leukocytes and the coagulation cascade, thus impairing oxygen delivery paradoxically [74–76].

Colloids have been used for acute fluid resuscitation in trauma, perioperatively and in critically ill patients, due to their longer intravascular persistence. Recent studies have shown no evidence of a significant mortality benefit from resuscitation with colloids [77–81]. In critically ill patients, the use of hydroxyethyl starch has been associated with AKI [77, 82]. However, this association has not been demonstrated in the surgical setting, namely after living donor hepatectomy, cardiac surgery, or gastroenterological surgery [83–85].

**Fig. 1:**
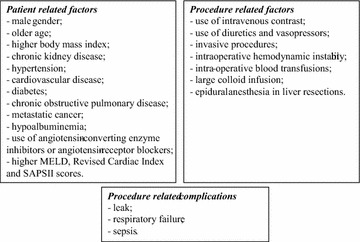
Risk factors for AKI after major abdominal surgery

Furthermore, patients who developed significant postoperative complications, such as leak, respiratory failure and sepsis, also have an increased rate of AKI [58, 59, 61, 62] (Fig. 1).

### Pathogenesis

The pathogenesis of postoperative AKI is complex and multifactorial. In this setting, we must consider not only the effects of fluid depletion, but also the neuroendocrine response to anesthesia and surgery itself [86, 87].

Fluid depletion includes the preoperative period as a result of the routine nil-by mouth regimens and the loss of fluid through concomitant pathology, and the perioperative period resulting from blood and intravascular fluid losses, insensible losses, and the so-called third space effect, through extravasation of fluid out of the vascular compartment. Mechanical ventilation of the intubated patient constitutes an additional mechanism for increased fluid loss during general anesthesia. The perioperative fluid requirements vary according to the extent of the surgical insult [86].

The renal response to hypoperfusion is afferent arteriole dilation and efferent arteriole vasoconstriction to maintain glomerular filtration in addition to neurohormonal responses as a means to expand the intravascular volume [57, 86, 87]. The increases in sympathomimetic hormones lead to renal cortical vasoconstriction, which is a compensatory attempt to redistribute blood flow to the renal medulla, but in fact causes ischemia of the medulla which is particularly vulnerable due to its elevated metabolic demand [57, 86, 87].

Most anesthetics cause peripheral vasodilatation and myocardial depression, also impairing kidney perfusion [86, 87]. The effect of the surgery results in both an increase in catabolic hormones and cytokines, leading to increased secretion of antidiuretic hormone, which will result in water retention. Increases in aldosterone, through activation of the renin–angiotensin system, associated with increased glucocorticoids cause sodium and water retention and potassium loss. Plasma renin activity is also elevated as a result of a decrease in circulating blood volume. Thus, adjustments in overall fluid and electrolyte homeostasis occur on account of impaired water excretion, impaired sodium excretion, and increased excretion of potassium [86].

Patients with long-term ACEI therapy have higher risk of postoperative renal dysfunction as a result of a loss of ability of the renin–angiotensin system to compensate for decreases in renal perfusion [86, 87].

Ischemic kidneys are more susceptible to continuing detrimental insults, such as, nephrotoxins and sepsis [86]. Nephrotoxins such as contrast media increase intrarenal vasoconstriction, decrease medullary blood supply and present the medullary nephrons with an increased osmotic load leading to an increased oxygen requirement in the presence of an already low tissue oxygen tension [88].

Nevertheless, in most cases, hemodynamic or toxic actions seem to be insufficient in the pathogenesis of AKI [89]. The role of nonhemodynamic factors, such as dysfunctional inflammatory cascades, oxidative stress, activation of proapoptotic pathways, differential molecular expression, and leukocyte trafficking, in AKI has been increasingly recognized [89, 90]. During abdominal surgery, a pro-inflammatory response is activated by the released endotoxin load from gut ischemia, impaired visceral perfusion, and portal endotoxaemia [91]. Furthermore, in the postischemic or reperfusion period there is further tubular injury caused by reactive oxygen species and tissue inflammation [90, 92]. The immune activation following AKI appears to negatively impact other organs [89].

## Outcomes

Various studies have verified the deleterious impact of AKI on the early outcomes of patients, namely longer lengths of hospital stay, increased healthcare costs, increased mortality and an increased likelihood of discharge to an extended care facility [46, 93–97]. Granting that AKI patients may have more comorbidities than non-AKI patients, these do not appear to account for all of the increased early mortality associated with AKI [3, 46, 97, 98]. Other factors should perhaps be regarded since even increases in SCr considered as minor lead to worse outcomes [88, 97, 98]. Accordingly, AKI has been progressively more thought of as part of a systemic disease with underlying mechanisms that cause multiorgan dysfunction including the kidney, which could help explain the decreased survival observed in AKI patients [87, 99].

An observational study by Grams et al. demonstrated an association between postoperative AKI after major surgery and longer lengths of stay (15.8 vs 8.6 days) and higher rates of 30-day hospital readmission (21 vs 13%) [48].

The association between a higher incidence of other postoperative complications, increased length of stay, higher healthcare costs and increased hospital readmissions and postoperative AKI related to major abdominal surgery has also been widely described. Lee et al. performed a retrospective analysis of 595 esophageal cancer surgery patients and established that the extent of hospital stay was significantly longer in patients with AKI [62]. In a retrospective review of 339 colectomies by Causey et al., AKI development was associated with a 5-day increase in hospital length of stay and nearly doubled the rate of other infectious complications (56 vs 30%) [61]. Tomozawa et al. reported that AKI after liver resection surgery was correlated with prolonged length of stay, and increased rates of artificial ventilation, need for reintubation, and requirement for renal replacement therapy [65]. In a retrospective study by Kim et al. gastric surgery patients with AKI had significantly longer hospital stay and higher prevalence of intensive care unit (ICU) admission after the operation (mean 18.7 vs 12.0 days, *P* < 0.001; 9.1 vs 1.2%, *P* < 0.001, respectively) [67].

The influence of postoperative AKI on higher in-hospital and 30-day mortality has also been demonstrated after major abdominal surgery. Kim et al. conducted a retrospective study of 4718 gastric surgery patients and reported that the in-hospital and 3-month mortality for patients with AKI were significantly higher than those for patients without AKI (3.5 vs 0.2%, *P* < 0.001; 3.8 vs 0.3%, *P* < 0.001, respectively), and moreover that the rate of in-hospital and 3-month mortality increased with the advancement in the stage of AKI, in a stepwise manner [67]. In a retrospective analysis of 642 liver resection patients by Tomozawa et al., AKI was associated with increased mortality (14.1 vs 2.3%, *P* < 0.0001) [65]. In a study by Teixeira, et al., 450 major abdominal surgery patients were retrospectively studied and postoperative AKI was independently associated with increased in-hospital mortality (20.8 vs 2.3%, *P* < .0001; unadjusted OR 11.2, 95% CI 4.8–26.2, *P* < .0001; adjusted OR 3.7, 95% CI 1.2–11.7, *P* = 0.024), furthermore there was a direct relationship between more severe AKI and increased in-hospital mortality [8]. O’Connor has also recently reported a 12.6-fold relative mortality risk in patients with postoperative AKI after major abdominal surgery [23].

Additionally, it is known that the detrimental effects of AKI persist after hospitalization, with greater risk of developing CKD and increased long-term mortality in AKI patients [20, 100, 101]. Progression to CKD results from an inadequate resolution of the acute insult following AKI, with persistent inflammation, increased transformation of pericytes into myofibroblasts in response to tubular injury, and consequent build-up of extracellular matrix and vascular rarefaction, leading to permanent scarring in renal structure and changes in renal function [102]. The risk of development or progression of CKD occurs in proportion to the severity of AKI [103]. The increased risk of proteinuria and hypertension and GFR decline described after AKI are known risk factors for cardiovascular disease, and may contribute to the decrement in survival observed among AKI survivors [104–107].

The long-term effect of AKI in postoperative patients has also been described. In a retrospective cohort study of 10,518 patients with AKI discharged after a major surgery, Bihorac et al. [20] reported that even small changes in creatinine level during hospitalization were associated with an independent long-term risk of death. Also, Grams et al. [48] performed an observational study of 3.6 million veterans submitted to major surgery and described an association between postoperative AKI and 1-year end-stage renal disease (0.94 vs 0.05%), and mortality (19 vs 8%), with more severe stage of AKI relating to poorer outcomes.

In a retrospective cohort of 390 major abdominal surgery patients, Gameiro et al. [108] demonstrated that AKI was independently associated with worse renal outcomes, comprising renal function decline and/or long-term need for dialysis (47.2 vs 22.0%, *P* < 0.0001), as well as with mortality after hospital discharge (47.2 vs 20.5%, *P* < 0.0001).

## Conclusion

AKI is a frequent occurrence following major abdominal surgery and is independently associated with both in-hospital and long-term mortality, as well as with a higher risk of progressing to CKD. Preventive strategies such as hemodynamics stabilization, fluid balance control, evasion of nephrotoxins, improved preoperative patient management (body weight reduction, hypertension, diabetes, cardiovascular and pulmonary disease control) and prevention/treatment of any postoperative complications encountered could potentially reduce postoperative AKI and thereby improve patient outcomes.

## Abbreviations

AKI: acute kidney injury
CKD: chronic kidney disease
RIFLE: Risk, Injury, Failure, Loss of kidney function and End-stage kidney disease
AKIN: Acute Kidney Injury Network
KDIGO: Kidney Disease: Improving Global Outcomes
SCr: serum creatinine
UO: urine output
ACEI: angiotensin-converting enzyme inhibitors
MELD: Model for end-stage liver disease
SAPS II: Simplified Acute Physiology Score
ICU: intensive care unit
KIM-1: kidney injury molecule-1
NGAL: neutrophil gelatinase-associated lipocalin
